# In Lower‐Grade Gliomas, the SPARC Family Exacerbates Prognosis by Influencing Immunity, Stemness, and Metabolism

**DOI:** 10.1002/cnr2.70307

**Published:** 2025-08-08

**Authors:** Qiaoying Peng, Wenxia Zhou, Ying Chen, Yong Cai

**Affiliations:** ^1^ Department of Operation Room (OR), The First People's Hospital of Huzhou Affiliated Hospital of Huzhou Teachers College Huzhou China

**Keywords:** EMT, GSC, LGG, methylation, SPARC

## Abstract

**Background:**

Involvement of the SPARC stromal protein family in crucial biological regulatory mechanisms is well‐documented. But understanding the consequences of imbalanced SPARC protein activity in lower‐grade glioma (LGG) is still emerging.

**Aims:**

Examining the clinical significance of SPARC proteins, researchers employed RNA‐seq data from diverse patient groups to gain insight. A novel SPARCScore was developed via LASSO regression analysis, leveraging data from the PanCanAtlas and MEXPRESS to shed light on the molecular mechanisms involved.

**Methods and Results:**

Our findings indicate that a majority of SPARC family proteins show atypical expression levels, correlating significantly with adverse outcomes in LGG. Our construction of an SPARCscore, indicative of the SPARC family's presence, revealed a direct correlation between a high SPARCscore and worsened tumor prognosis, irrespective of radiotherapy or chemotherapy treatments. The SPARCScore risk groups showed distinct drivers: PIK3CA predominantly influenced the low‐risk category, whereas EGFR was a key factor in the high‐risk group. High SPARCScore tumors exhibited a mutation profile similar to glioblastoma, marked by reduced methylation and diverse glioma stem cells (GSC). Conversely, the low SPARCScore tumors were characterized by increased methylation and limited GSC variety. Furthermore, the high‐SPARCScore group was notable for its pronounced inflammatory and extracellular matrix signatures, along with activated metabolic pathways. These patterns were closely linked to prognosis.

**Conclusion:**

In essence, this research highlights the significance of SPARC proteins in LGG, offering insights into promising avenues for targeted therapy.

**Abbreviations**
BER: increased base excision repair
CAFs: cancer‐associated fibroblasts
CNV: copy number variations
DDR: DNA damage response
DS: double‐strand break processes
ECM: extracellular matrix components with a notable increase in the high‐SPARCScore group (Figure 6A)
EMT: epithelial‐mesenchymal transition
FA: Fanconi anemia
GBM: glioblastoma
GSC: glioma stem cells
HR: homologous recombination
LGG: lower‐grade glioma
LOH: loss of heterozygosity
MET: mesenchymal‐to‐epithelial transition
MMR: mismatch repair
NER: nucleotide excision repair
NHEJ: non‐homologous end joining
OS: overall survival
TLS: translesion synthesis
TME: tumor microenvironment
WHO: World Health Organization

## 1 Introduction

Gliomas are the leading malignant brain tumors, accounting for 75% of all central nervous system tumors. In China, they represent 31.1% of brain tumors in people aged 20–59 [1]. The survival rates for different grades of gliomas are poor, with an average survival period of around 6.5 years for WHO grade II gliomas, 3.1 years for grade III, and only 1.2 years for glioblastoma (GBM). There is an urgent need for new treatment strategies for gliomas, as there has been little progress in recent years in China, which has a high incidence and mortality rate for brain tumors [2]. Immunotherapy, which has been revolutionary in cancer treatment, has not been effective in gliomas due to their unique tumor microenvironment (TME) [3, 4]. Targeting therapeutic agents within the glioma TME is a promising new direction for research and treatment.

Tumor microenvironments comprise tumor cells, immune and stromal cells, extracellular matrix, vascular systems, and other molecules. In brain tissues, the extracellular matrix constitutes about 20% of the volume, distinct from other tissues mainly composed of collagen and elastin, as brain tissues primarily consist of glycosaminoglycans, hyaluronic acid, and lectican proteoglycans. In gliomas, the density of the extracellular matrix positively correlates with tumor grading [5]. Additionally, studies have shown that biomechanical changes in the extracellular matrix of brain gliomas affect tumor cell migration. The harder the texture of the extracellular matrix, the more likely the tumor is to migrate outward, further regulating the expression of adhesion genes and tumor cell adhesion [6]. Therefore, further exploration of the extracellular matrix structure remodeling and potential molecular mechanisms in gliomas is necessary.

The SPARC family of proteins consists of SPARC, SPARC‐like 1 (SPL1), SMOC 1 and 2, SPOCK1, 2, 3, and Follistatin‐like 1 (Fstl‐1). These proteins share three conserved domains: N‐terminal, follistatin‐like, and C‐terminal. The SPARC family is implicated in several cancers [7]. Fstl1, a member of the family, is elevated in high‐grade gliomas and contributes to tumor growth via the BMP4/Smad1/5/8 pathway [8]. Higher Fstl1 levels are linked to worse prognosis [9]. Additionally, increased SMOC1 expression has been observed in LGG tissues, with high SMOC1 levels correlating with reduced patient survival [10]. Despite the importance of the SPARC family in gliomas, research into their roles in LGG is ongoing, and their specific functions are not yet fully understood.

This investigation seeks to thoroughly examine the roles and underlying mechanisms of the SPARCs within LGG. It started by analyzing the unique expression patterns of SPARC family proteins in LGG. Then, it investigated the clinical significance of these proteins, including their association with patient survival, clinical characteristics, molecular classification, and treatment response. A prognostic SPARC family risk score was developed to predict survival and assess treatment effectiveness. The study further investigated the underlying mechanisms of the SPARC family's impact on LGG prognosis, including their interactions with genomic and epigenomic factors, their influence on glioma stem cells (GSCs), and their role in the immune response. The research uncovers the critical roles and mechanisms of the SPARC family in LGG, providing new insights and potential therapeutic targets for personalized glioma management.

## 2 Materials and Methods

### 2.1 Data Collection

RNA‐seq data for LGG and associated patient information were obtained from The Cancer Genome Atlas (TCGA). RNA‐seq data from normal brain tissue, focusing on the cerebral cortex, were obtained from the Genotype‐Tissue Expression (GTEx) database. Additional clinical information, genomic characteristics, and molecular classifications were obtained from PanCanAtlas reports and the MEXPRESS database.

### 2.2 Analyzing Expression and Survival

Differences in expression levels were investigated using the “limma” tool in R between 207 typical cerebral cortex specimens and 529 LGG samples from TCGA, with the “pheatmap” tool in R used to create a heatmap representing the altered expression patterns [11]. Survival data from 506 TCGA‐LGG patients were utilized for survival analysis, employing the “surv_cutpoint” function to determine optimal groupings, and the “survival” and “survminer” packages for the analysis.

### 2.3 SPARCscore Development and Assessment

The SPARCscore was devised using “LASSO” regression over 1000 iterations to create a risk score based on the SPARC family. The analytical method of LASSO regression, on the other hand, by introducing a penalty term, can change the weights of small elements in the coefficient vectors to 0, and filter out the features that have the greatest predictive power for the target variable among the non‐zero coefficients. The score was computed using a specific formula incorporating regression coefficients and gene expression levels. During SPARCscore development, a random selection of 70% samples was allocated to the training dataset, while the remaining samples were set aside for testing purpose. The risk curve was plotted using “pheatmap,” and the “ComplexHeatmap” to visualize the correlation between SPARCscore and medical characteristics. A nomogram that includes SPARCscore and other clinical factors, along with calibration plots, was created using “rms”.

### 2.4 Single‐Sample Gene‐Set Enrichment

The “GSVA” package facilitated ssGSEA, utilizing 29 immune signatures to assess immune functions and pathways [12]. This analysis incorporated various gene sets related to metabolism, DNA damage response (DDR), ECM components, immunogenic cell death, and epithelial‐mesenchymal transition (EMT).

### 2.5 Genetic Mutation, Epigenetic Data and Stemness Indices

Using TCGA mutation data through the “maftools package” using “mafComapre” function to detect the difference between the two cohorts; The “oncoplot” function was used to draw a waterfall map of gene mutation frequency grouped by SPARCScore. Forest plots for comparison of gene mutation differences were plotted using the “forest plots” function. The oncodrive gene bubble map was calculated using the “Oncodrive” function. And the “OncogenicPathways” function shows the enrichment of oncogenic signaling pathways.

Methylation subtypes and GSCs signatures were identified based on existing literature and single‐cell sequencing data, respectively [13, 14]. Two indices of stemness were calculated to reflect gene expression and epigenetic features of stem cells, using the “ELMER” package for network reconstruction and “OCLR” for feature identification [15].

### 2.6 Tumor Microenvironment Analysis

Data on pan‐cancer immunophenotypes, leukocyte fractions, immune molecules, and antigenic peptide loads were sourced from published studies. The “CIBERSORT” and “xcell” packages quantified TME immune and stromal components from expression profiles. “TIDE” database was employed to evaluate immune dysfunction, exclusion, and response to immunotherapy.

### 2.7 Statistical Methods

Data analysis was conducted using R software, version 4.0.2. For comparing two sets, the Wilcoxon test was applied, and the Kruskal‐Wallis test was used for groups exceeding two. Kaplan–Meier methodology was employed to plot survival curves, with the Log‐rank test determining survival disparities. Statistical significance was established at a *p*‐value threshold of 0.05.

## 3 Results

### 3.1 Evaluating SPARC Proteins in LGG

**FIGURE 1:**
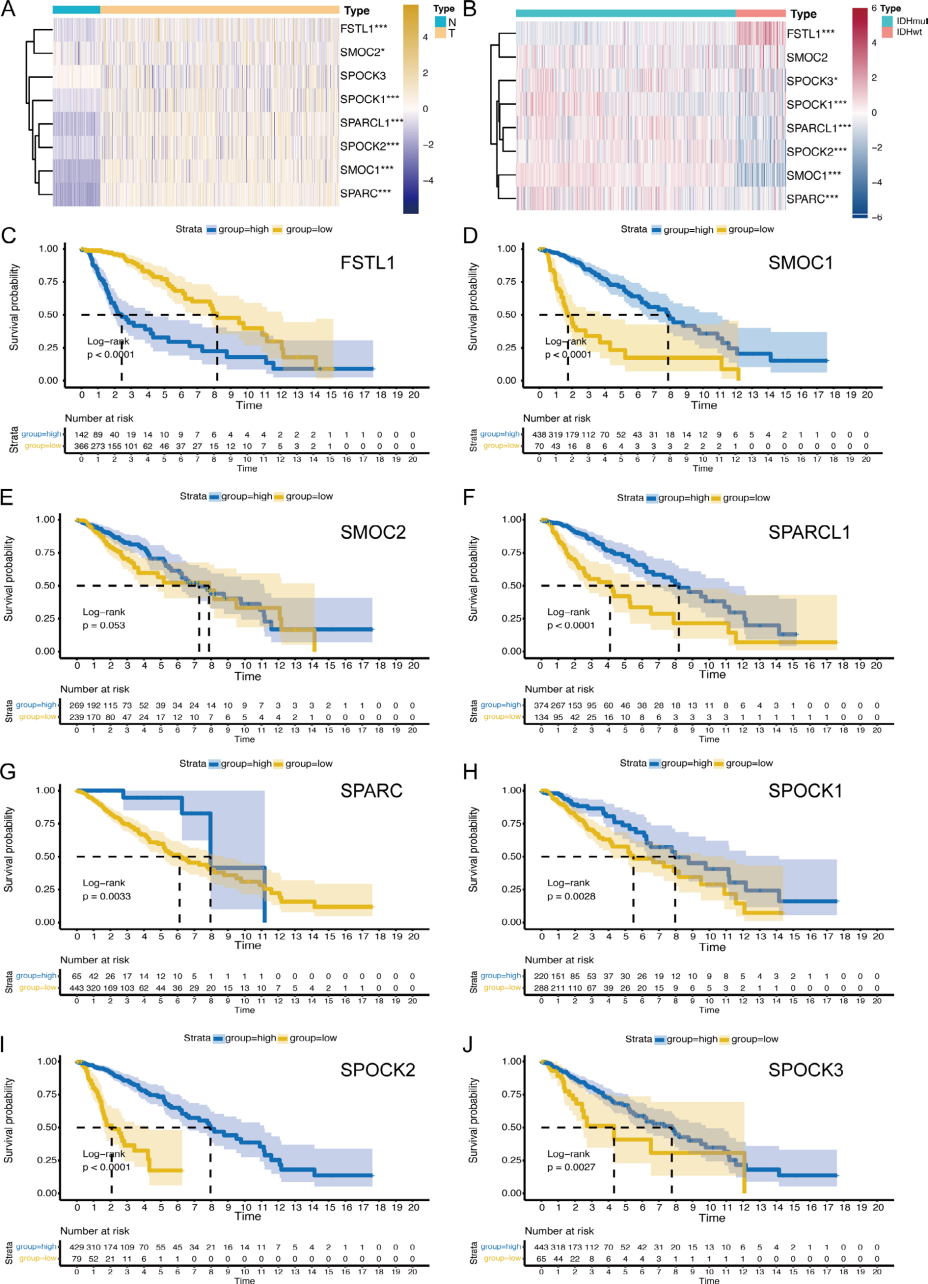
Differential expression and clinical prognostic value of SPARC family in normal brain tissue and low‐grade gliomas (TCGA). (A) Differential expression of SPARC family genes in normal brain tissue and LGG. (B) Differential expression of SPARC family genes in IDH‐mut and IDH‐wild LGG. (C–J) Survival analysis of high and low expression groups of SPARC family genes in LGG using the method of optimal cutoff values. *p < 0.05, **p < 0.01, ***p < 0.001.

To explore the dysregulation of SPARC proteins in LGG, we analyzed gene expression differences between LGG and normal brain tissue. Our findings, detailed in Figure 1A, reveal a significant variance in SPARC protein levels, particularly in LGG. Notably, FSTL1, SMOC1, SMOC2, SPOCK1, SPOCK2, SPARC, and SPARCL1 exhibited higher expression in LGG, while SPOCK's expression was comparably lower but not significantly different. Further, IDHwt subgroups showed elevated FSTL1 expression compared to IDHmut groups, with other proteins like SMOC1 and SPARC also more pronounced in IDHmut (Figure 1B). Gene expression comparisons between the three groups of normal brain tissue, IDHmut, and IDHwt are shown in Figure S1. Subsequently, assessing the impact of SPARC protein levels on LGG prognosis, we examined survival rates linked to these genes in the TCGA dataset (Figure 1C–J). Analysis distinguished SPARC genes into high‐exp and low‐exp categories, revealing a strong survival correlation for most, except SMOC2. Specifically, higher expression of SMOC1, SPOCK1, SPOCK2, SPARC, and SPARCL1 correlated with extended patient survival, in contrast to FSTL1, which indicated reduced survival times. These observations underscore the role of each SPARC protein in LGG.

### 3.2 Developing and Validating SPARCScore

**FIGURE 2:**
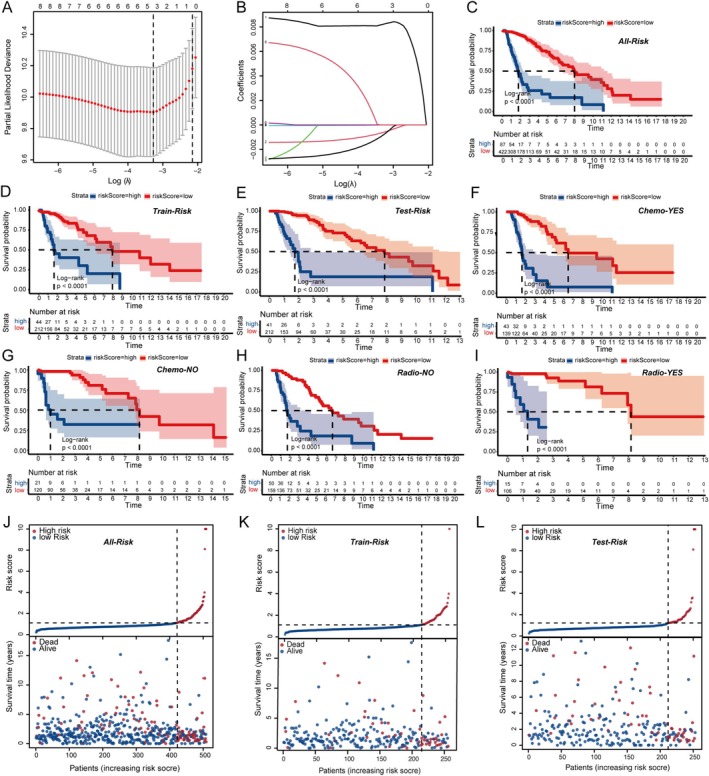
Construction and validation of the SPARCscore risk prediction model. (A, B) The minimum penalty coefficient was calculated by Lasso regression. (C–E) Survival analysis of SPARCscore‐high risk and SPARCscore‐low risk groups in the full data set (C), train set (D), and test set (E). (F–I) Survival analysis was performed in SPARCScore high and low risk groups with chemotherapy (F) or without chemotherapy (G), with radiotherapy (H) or without radiotherapy (I). (J–L) Risk curve of SPARCScore for the full set (J), train set (K) and test set (L) were plotted using the TCGA glioma cohort. *p < 0.05, **p < 0.01, ***p < 0.001.

To forecast LGG survival, we employed LASSO COX regression that considers SPARC family gene interrelations. The dataset was split into two sets, train set and test set to fit the model. Key SPARC genes were pinpointed by the LASSO‐COX model, as shown in Figure 2A, where the optimal penalty coefficient was determined to be −3.3. This analysis yielded three genes with significant coefficients (Figure 2B). The value of SPARCScore is determined by the sum of the expression of the three genes multiplied by their coefficients, leading to the creation of the SPARCScore. The coefficients assigned were 0.00823071186429766 for FSTL1, −0.000594413967440571 for SMOC1, and −0.000789561213511027 for SPOCK2. We categorized the cohort into a high SPARCScore risk subgroup and a low SPARCScore risk subgroup using the optimal cutoff value of 2.05482168652365 computed via the surv‐cutpoint function (Figure 2C–E), and we noted a correlation between elevated SPARCScores and decreased survival rates. This trend persisted across different treatment modalities, with both chemotherapy and radiotherapy groups showing similar prognostic patterns when segmented by SPARCScore (Figure 2F–I). Additionally, the high‐risk group exhibited a greater mortality rate, as illustrated by the SPARCScore distribution and survival data (Figure 2J–L).

### 3.3 Analyzing SPARCScore and Clinical Characteristics in LGG

**FIGURE 3:**
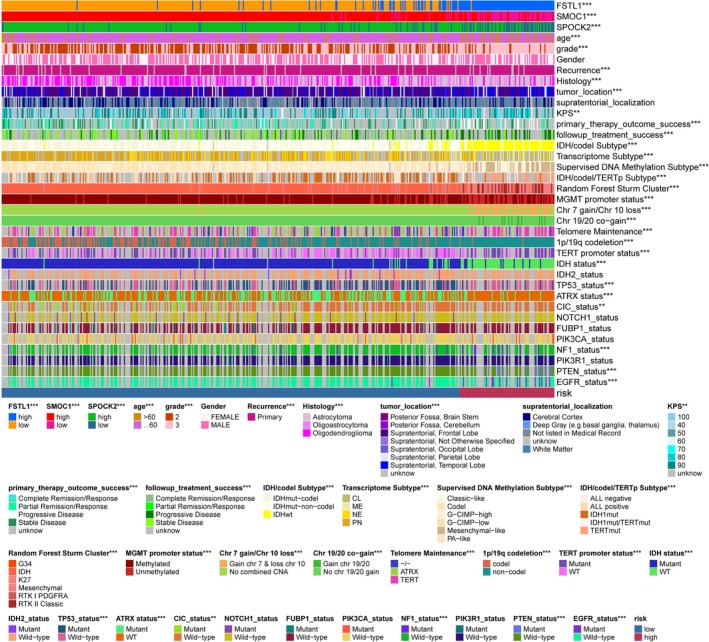
Correlation heatmap of the SPARCScore versus common clinical traits, therapeutic response, and molecular characteristics in the TCGA glioma cohort. *p < 0.05, **p < 0.01, ***p < 0.001.

Our study in the TCGA LGG cohort investigated the association of SPARCScores with a range of tumor characteristics, including clinical features, molecular subtypes, and response to standard treatments. We observed distinct trends: older patients, those with high‐grade tumors, and astrocytic involvement predominantly fell into the high SPARCScore group. This group also had a higher incidence of disease progression post‐initial treatment and at follow‐up. Among molecular subtypes, patients with high SPARCScores were characterized by a higher prevalence of IDH1, TP53, and ATRX wild types, and mutations in NF1, PTEN, and EGFR. They frequently exhibited chromosomal alterations such as 19/20 co‐gain, 7 gain with 10 loss, and 1p19q co‐deletion. Additionally, they tended to have TERT‐maintained telomeres, TERT promoter mutations, and unmethylated MGMT promoters. The high SPARCScore was notably common in IDH wild‐type, classic‐like, mesenchymal‐like, hypo‐methylated, and TERT mutation subtypes. Notably, high SPARCScores were prevalent in IDH wild‐type, classic‐like, mesenchymal‐like, hypo‐methylated, and TERT mutation molecular subtypes. These findings, illustrated in Figure 3A, suggest a strong correlation between high SPARCScores and poorer prognosis in LGG.

### 3.4 Investigating Genomic and Epigenetic Profiles in SPARCScore Subgroups of LGG

**FIGURE 4:**
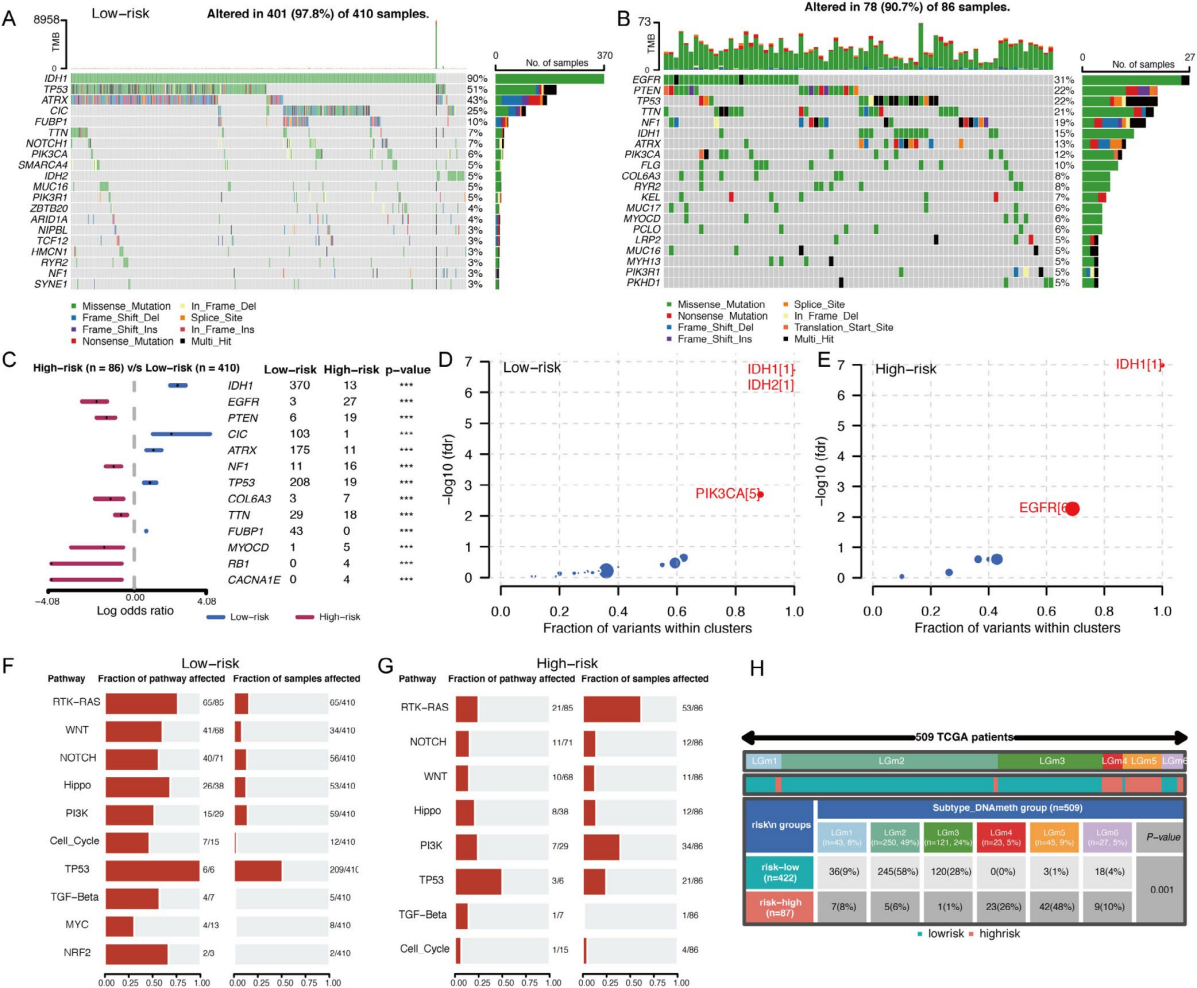
Mutational landscape, Pan‐glioma methylation subtypes, and affected pathways comparison in TCGA glioma cohort based on SPARCScore. (A, B) Somatic mutation waterfall plots were created based on low‐risk (A) and high‐risk groups (B). (C) Forest plot showed the differential mutation patterns in the high‐risk and low‐risk groups. (D, E) Bubble plot showed the driver genes of low‐risk (D) and high‐risk groups (E). (F, G). Fraction of pathway and samples affected between low‐risk (F) and high‐risk groups(G). (H) Pan‐glioma methylation subtypes in high‐risk and low‐risk groups.

We used the “Maftools” package to differentiate mutation frequencies, driver mutations, and oncogenic pathways in LGG, and the samples were categorized into two groups according to their SPARCScore. As shown in Figure 4A,C, the somatic mutation waterfall plots showed the genes with the highest mutation frequency in each group. The gene with the highest mutation frequency in the low SPARCScore group was IDH1, while the EGFR mutation was the most frequent in the high SPARCScore group. Figure 4C more clearly demonstrated the comparison between the different mutated genes between the two groups, and we found that there were significantly different mutation patterns in the two groups, with the high SPARCScore group dominated by mutations in genes such as EGFR, PTEN, and NF1, while in the low SPARCScore group, IDH1, CIC, and ATRX mutations were more prominent. In addition to the analysis by gene mutation frequency, we also analyzed driver mutations to identify mutations with low mutation frequency but functionally important mutations, and we found that IDH mutations remained a common starting point in both subgroups, whereas PIK3CA mutations defined the low‐SPARCScore group, and EGFR mutations were the driver mutations in the high‐SPARCScore group. In addition, our analysis also showed that the oncogenic pathways of the two groups were also differentiated by (Figure 4F,G). In summary, there was significant inter‐tumor heterogeneity between the low and high SPARCScore groups, which underlies the differences in tumor biological behaviors and the differences in prognosis.

Furthermore, we delved into the epigenetic differences using methylation subtype analysis in pan‐glioma, as illustrated in Figure 4H. The LGm1–3 group represents the hypermethylated group and the LGm4–6 group is the hypomethylated group. The high‐SPARCScore subgroup showed more distribution in LGm4 and LGm5, indicative of genome‐wide hypo‐methylation and often IDH wild‐type, while the LGm2 and LGm3 groups, associated with genome‐wide hyper‐methylation and IDH mutations, were more prevalent in the low‐SPARCScore subgroup.

### 3.5 Sparcscore's Link to LGG Cell Stemness, Genomic Instability, and Metabolic Alterations

**FIGURE 5:**
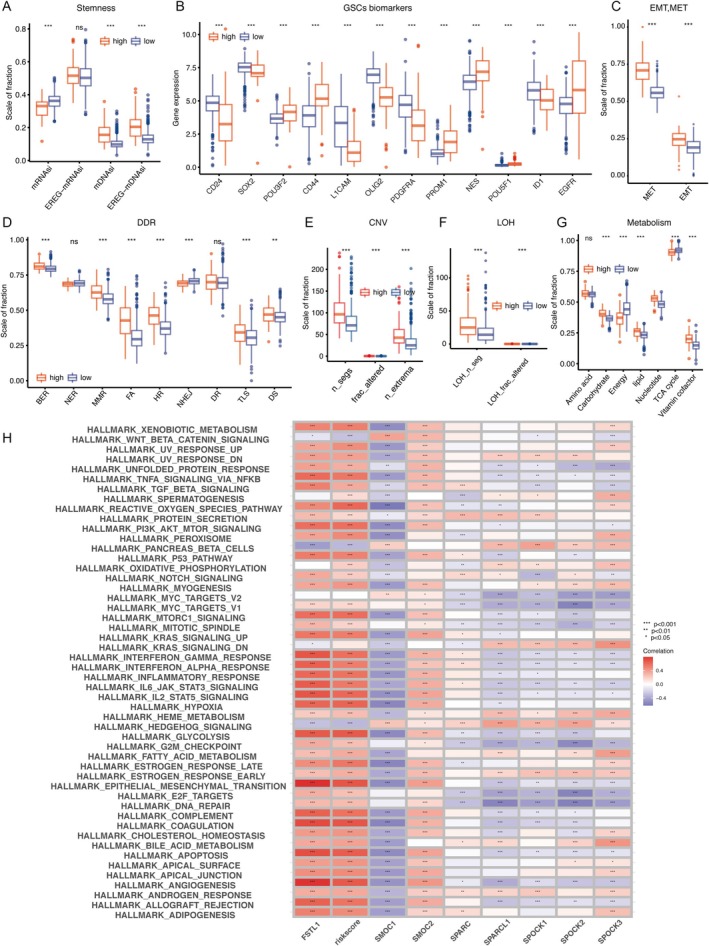
Relationship between SPARCScore and tumorigenesis. (A–C) Differences in stemness index (A), markers of glioma stem cells (B), and EMT versus MET phenotype (C) between high risk and low risk subgroups. (D–G) Differences in DNA damage repair pathways (D), copy number variation burden (E), loss of heterozygosity (F) and metabolism level (G) between High‐risk and Low‐risk SPARCScore groups. (H) Correlation between SPARC family genes, SPARC family genes, and hallmark signal pathways. *p < 0.05, **p < 0.01, ***p < 0.001. ns, no significance.

Stem cell characteristics are pivotal in cancer development. We assessed the association between SPARCScore and glioma cell stemness using indices like mRNAsi and its epigenetically regulated counterpart, EREG‐mRNAsi, as well as DNA‐based indices (mDNAsi and EREG‐mDNAsi), as shown in Figure 5A. The high‐SPARCScore group demonstrated increased mDNAsi and EREG‐mDNAsi, indicating enhanced stemness. Conversely, mRNAsi exhibited an inverse relationship. mRNAsi was lower in the high‐SPARCScore group and higher in the low‐SPARCScore group, which correlated with the enrichment of IDH mutations in the low‐SPARCScore group. We also examined glioma stem cell (GSC) markers, including cell surface proteins and transcription factors. Most of the GSC markers showed a clear bias toward one of the four GBM cell states: CD24 was highest in npc‐like cells, CD133 was highest in opc‐like cells, EGFR was highest in ac‐like cells, and CD44 was highest in mes‐like cells. Among TFs and lineage markers, NES showed a significant bias toward ac‐like cells. High‐SPARCScore tumors expressed higher levels of POU3F2, CD44, PROM1, NES, POU5F1, and EGFR, suggesting a prevalence of AC‐like, OPC‐like, and MES‐like cells, while NPC‐like cells were more common in the low‐SPARCScore group (Figure 5B).

We further investigated the role of epithelial‐to‐mesenchymal transition (EMT) and mesenchymal‐to‐epithelial transition (MET) in stem cell dynamics, with the high‐SPARCScore subgroup showing enhanced EMT and MET activity, particularly MET, indicating a propensity for cellular state transitions (Figure 5C). Genomic stability is another cancer hallmark. We analyzed DNA damage response (DDR) pathway functionality between SPARCScore subgroups (Figure 5D), finding that the high‐SPARCScore group exhibited increased base excision repair (BER), mismatch repair (MMR), Fanconi anemia (FA), homologous recombination (HR), translesion synthesis (TLS), and double‐strand break (DS) processes, while nucleotide excision repair (NER) and non‐homologous end joining (NHEJ) were diminished. This altered DDR functionality correlated with higher copy number variations (CNV) and loss of heterozygosity (LOH) in the high‐SPARCScore group, as depicted in Figure 5E,F.

Metabolic dysregulation was also scrutinized, revealing significant differences in carbohydrate, lipid, nucleotide, and vitamin coenzyme metabolism between high and low SPARCScore groups, with the former showing reduced energy metabolism and tricarboxylic acid cycle activity (Figure 5G). Lastly, we examined the connection between SPARCScore, SPARC family genes, and hallmark signaling pathways. High SPARCScores and FSTL1 were positively correlated with several pathways, particularly EMT, while SMOC1 showed an inverse relationship (Figure 5H).

### 3.6 Sparcscore's Relationship With TM's Stromal and Immune Elements in LGG

**FIGURE 6:**
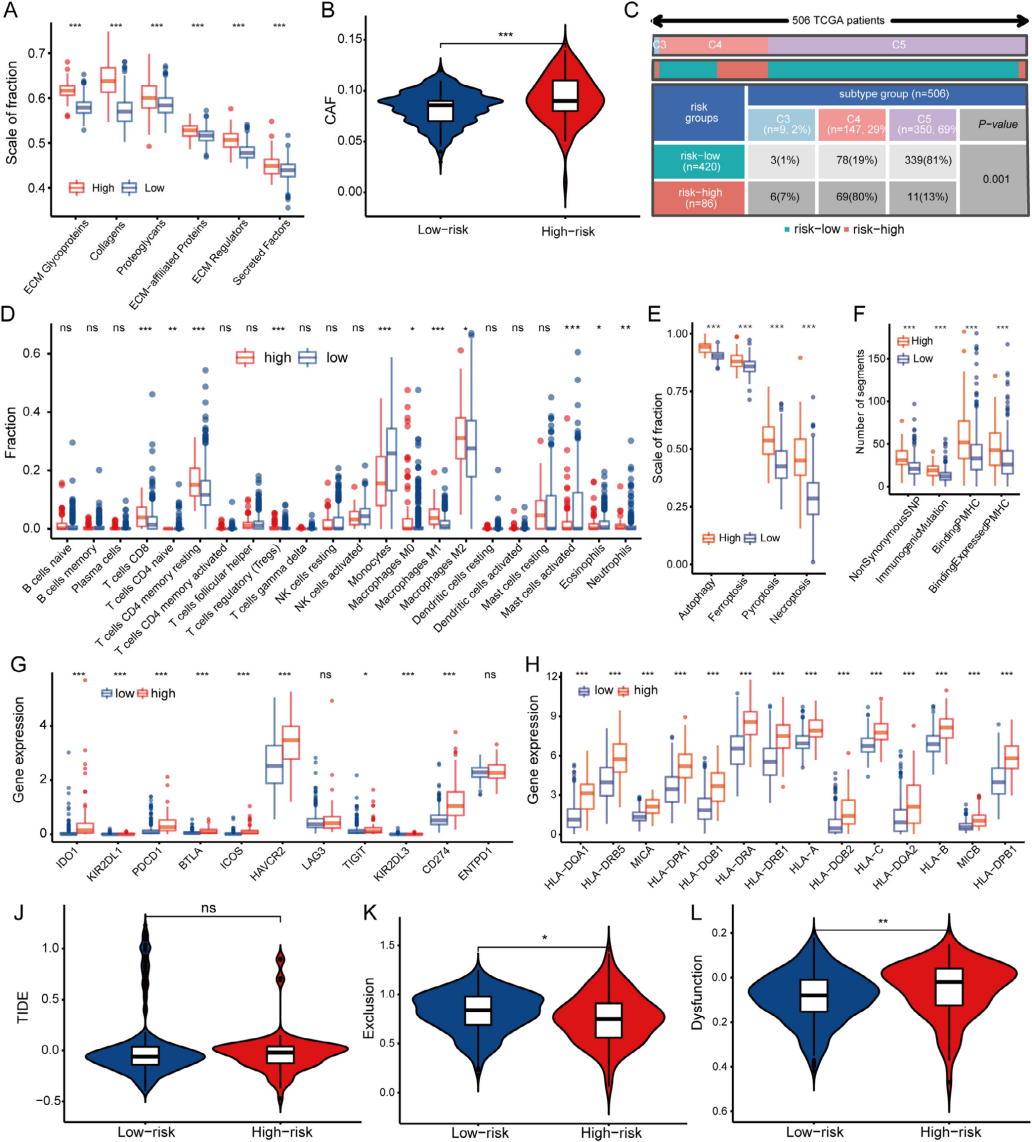
Extracellular matrix of LGG and immune characteristics of LGG based on SPARCScore. (A, B) Difference between extracellular matrix components (A) and cancer‐associated fibroblasts (B) in high‐risk and low‐risk subgroups. (C, D). Proportion of pan‐glioma immune subtypes (C) and immune cells infiltrating (D) in tumor between high‐risk and low‐risk groups. (E, F) Immunogenic death (E) in glioma TME based on SPARCScore, antigenic peptide‐major histocompatibility complex (F) of high‐risk and low‐risk groups. (G, H) Differences in the expression of immune checkpoints (G) and major histocompatibility complexes (H) in TME between high‐risk and low‐risk groups. (I, K) Differences in immune escape (I), immune exclusion (J), and immune dysfunction (K) between high and low risk groups. For all experiments, mean rank, *p < 0.05, **p < 0.01, ***p < 0.001. ns, no significance.

We examined the link between SPARCScore and the stromal makeup of LGG's tumor microenvironment (TME). Our analysis revealed a positive correlation between SPARCScore and the levels of extracellular matrix (ECM) components, with a notable increase in the high‐SPARCScore group (Figure 6A). Cancer‐associated fibroblasts (CAFs) were also more abundant in this subgroup (Figure 6B).

When assessing the immune landscape of LGG, of these, c1 is the (wound‐healing type), characterized by elevated angiogenic gene expression, a high proliferation rate, and adaptive immune infiltration with a predisposition to Th2 cells. c2 is the (IFN‐γ dominant type) characterized by the highest M1/M2 macrophage polarization and strong CD8 signaling. c3 is the (inflammatory type) displaying elevated Th17 and Th1 genes and low to moderate tumor cell proliferation. c4 is the (lymphocyte‐depleted type), displaying a more prominent macrophage profile with suppressed Th1 and high M2 responses. c5 is the (immunologically quiet type) displaying the lowest lymphocyte profile and highest macrophage profile. c6 is the (TGF‐β dominant type) showing the highest TGF‐β features and high lymphocytic infiltration, with uniform distribution of type I and type II T cells. Distinct immunophenotypes emerged, with C4 being more prevalent in the high‐SPARCScore subgroup and c5 in the low‐SPARCScore subgroup (Figure 6C). Immune cell infiltration analysis showed a higher presence of CD4 memory resting T cells and CD8 T cells in the high‐SPARCScore group, while monocytes were more prominent in the low‐SPARCScore group (Figure 6D).

Further investigation into immune antigenicity and presentation revealed that the high‐SPARCScore group exhibited increased levels of immunogenic cell death markers, including autophagy, ferroptosis, pyroptosis, and necroptosis (Figure 6E). This subgroup also showed elevated expression of MHC‐associated genes and immunogenic mutations (Figure 6F). Additionally, immune checkpoints such as TIM‐3 (HAVCR2) and PD‐1 (CD274), along with immunosuppressive gene expression, were higher in the high‐SPARCScore group, suggesting a complex immune environment with both activation and suppression (Figure 6G,H). To understand immune evasion tactics, we utilized the TIDE database to analyze immune escape processes. The high‐SPARCScore group was associated with increased immune dysfunction, while the low‐SPARCScore group showed higher immune exclusion, indicating compromised immune activity in the former (Figure 6J–L).

## 4 Discussion

Initially, we confirmed the distinct expression patterns and prognostic value of SPARC proteins in low‐grade gliomas (LGG). We then introduced the novel SPARCScore, derived from the SPARC family, which proved to be a pivotal tool for predicting patient outcomes, classifying tumor subtypes, and guiding therapeutic decisions. Our subsequent investigations delved into the genomic and epigenetic landscapes, as well as the tumor microenvironment (TME) features, of the High‐SPARCScore and Low‐SPARCScore patient cohorts. We discovered that the High‐SPARCScore group exhibited a genetic profile reminiscent of glioblastoma multiforme (GBM), marked by increased cellular stemness, genomic instability, inflammation, extracellular matrix (ECM) presence, metabolic dysregulation, and a generally unfavorable prognosis. In conclusion, our extensive multi‐omic analysis of the SPARC family highlighted its significant influence on the stromal components in LGG, offering novel insights for enhancing LGG treatment strategies.

To investigate the expression patterns of SPARC family proteins in low‐grade gliomas (LGG), we compared gene expression profiles between LGG and normal cortex samples. As shown in Figure 1A, LGG exhibited pronounced alterations in SPARC family expression. FSTL1, SMOC1, SMOC2, SPOCK1, SPOCK2, SPARC, and SPARCL1 were generally upregulated, with SPOCK3 expression showing a non‐significant decrease. IDH wild‐type (IDHwt) LGGs had higher FSTL1 levels than IDH mutant (IDHmut) cases, while IDHmut LGGs displayed increased expression of SMOC1, SPOCK1, SPOCK2, SPOCK3, SPARC, and SPARCL1. Except for SMOC2, SPARC family gene expression significantly correlated with survival, where higher SMOC1, SPOCK1, SPOCK2, SPARC, and SPARCL1 levels were predictive of improved survival, and high FSTL1 expression was associated with reduced survival.

Increased SMOC1 expression is detected in brain tumors and particularly in LGG, correlating with improved survival [10]. Research has also noted a relationship between methylation patterns at CpG sites targeting SMOC1, KCNA4, SLC25A21, and UPP1, and the molecular characteristics of glioma, such as IDH mutations and 1p/19q co‐deletions, which aligns with our findings [16]. Furthermore, studies have consistently reported reduced SPOCK2 expression in high‐grade gliomas [17]. SPOCK2 is involved in countering the inhibitory effects of the testican family on matrix metalloproteinases [18]. Overexpression of FSTL1 has been identified as an indicator of an unfavorable prognosis in GBM patients [19], with research indicating that Fstl1 stimulates glioma growth via the BMP4/Smad1/5/8 signaling pathway [9]. The Fstl1/DIP2A/MGMT pathway is linked to temozolomide resistance in glioblastoma, with increased Fstl1 expression contributing to resistance, while decreased expression enhances drug sensitivity [8]. Collectively, these findings from existing literature corroborate our study's outcomes.

Research indicates that the tumor microenvironment (TME) of brain tumors is modulated by their molecular traits [13]. Our analysis revealed a predominance of the C5 subtype within the Low‐SPARCScore group. Notably, IDH mutations, which are prevalent in 80% of the C5 subtype [20], are known to mitigate leukocyte chemotaxis, thereby reducing the presence of tumor‐associated immune cells and improving prognosis [21]. This aligns with the characteristics observed in the Low‐SPARCScore group. Additionally, deletions on chromosomes 1p and 19q, which affect genes like TNFRSF9, VTCN1, and TGFB1, are linked to lower levels of lymphocyte infiltration (LF), resonating with the known influence of TGF‐β on immune cell recruitment [22]. This further supports the molecular‐TME consistency within the Low‐SPARCScore group. Conversely, our data indicated a marked increase in macrophage populations in the High‐SPARCScore group. Considering that macrophages constitute about 30% of immune cells in brain tumors [23] and their abundance is negatively associated with patient survival and positively with tumor grade [24], this is a significant finding. NF‐1 deficiency in IDH‐wildtype gliomas, often seen in the mesenchymal subtype, is known to attract more macrophages. In addition to macrophages, the High‐SPARCScore subgroup exhibited an elevated presence of MDSC and CD8 T cells. The augmented CD8 T population seemed to be unrelated to particular molecular types, instead being linked to a hypermutated profile [13], which could lead to the generation of more neoantigens detectable by T cells, corroborating our results.

Our study, while comprehensive, has certain limitations. Firstly, the functional class scoring (FCS) method was employed for phenotypic and pathway quantification. FCS, analyzing pathways individually, might lead to overemphasized enrichment in certain pathways due to gene overlap; genes often participate in multiple pathways. Secondly, our research is focused at the transcriptome level, emphasizing the importance of proteins as the primary functional units. This approach necessitates further investigation into the realms of post‐transcriptional and post‐translational modifications to fully understand their impact. Lastly, our study primarily establishes correlational evidence. Therefore, it is essential to combine these findings with basic experimental research to validate causal relationships and explore the direct interactions both upstream and downstream.

## 5 Conclusions

Our work demonstrates the clinical relevance of the SPARCs in LGG and introduces the risk scoring model grounded in the SPARC family to assist with clinical decisions. Furthermore, we investigated the role of SPARCs in LGG's biological processes and identified connections between SPARCs and factors such as genetic alterations, increased inflammation, elevated ECM production, stem cell properties, metabolic irregularities, immune evasion, and tumor aggressiveness. The results highlight the significant and unique research contribution of SPARC proteins to our understanding of LGG.

## Author Contributions

**Qiaoying Peng:** conceptualization (equal), data curation (equal), funding acquisition (equal), investigation (equal), methodology (equal), project administration (equal), resources (equal), software (equal), supervision (equal), visualization (equal), writing – original draft (equal), writing – review and editing (equal). **Wenxia Zhou:** data curation (equal), investigation (equal), methodology (equal). **Ying Chen:** conceptualization (equal), investigation (equal), methodology (equal), visualization (equal). **Yong Cai:** investigation (equal), methodology (equal), writing – original draft (equal).

## Conflicts of Interest

The authors declare no conflicts of interest.

## Supporting information


**Figure S1.** Differential expression of SPARC family genes in normal brain tissue, IDH‐mutant and IDH‐wild‐type gliomas. Statistical analysis was performed using the Kruskal‐Wallis test. *p < 0.05, **p < 0.01, ***p < 0.001. ns, not significance.

## Data Availability

The raw data and clinical data can be found at GDC (https://portal.gdc.cancer.gov/), CGGA (https://www.cgga.org/), GTEx (https://www.gtexportal.org) and the Pancancer Atlas publication page (https://gdc.cancer.gov/about‐data/publications/pancanatlas). Additional data resources for this manuscript are at https://gdc.cancer.gov/about‐data/publications/panimmune. The datasets used in this study, which are available to the public, can be accessed through the website detailed in the Supporting Information and Methods section.
