# Supplementary figures and images for: In Lower‐Grade Gliomas, the SPARC Family Exacerbates Prognosis by Influencing Immunity, Stemness, and Metabolism
Source: Cancer Rep (Hoboken). 2025 Aug 8;8(8):e70307. doi: 10.1002/cnr2.70307 (PMC12334845; doi:10.1002/cnr2.70307)

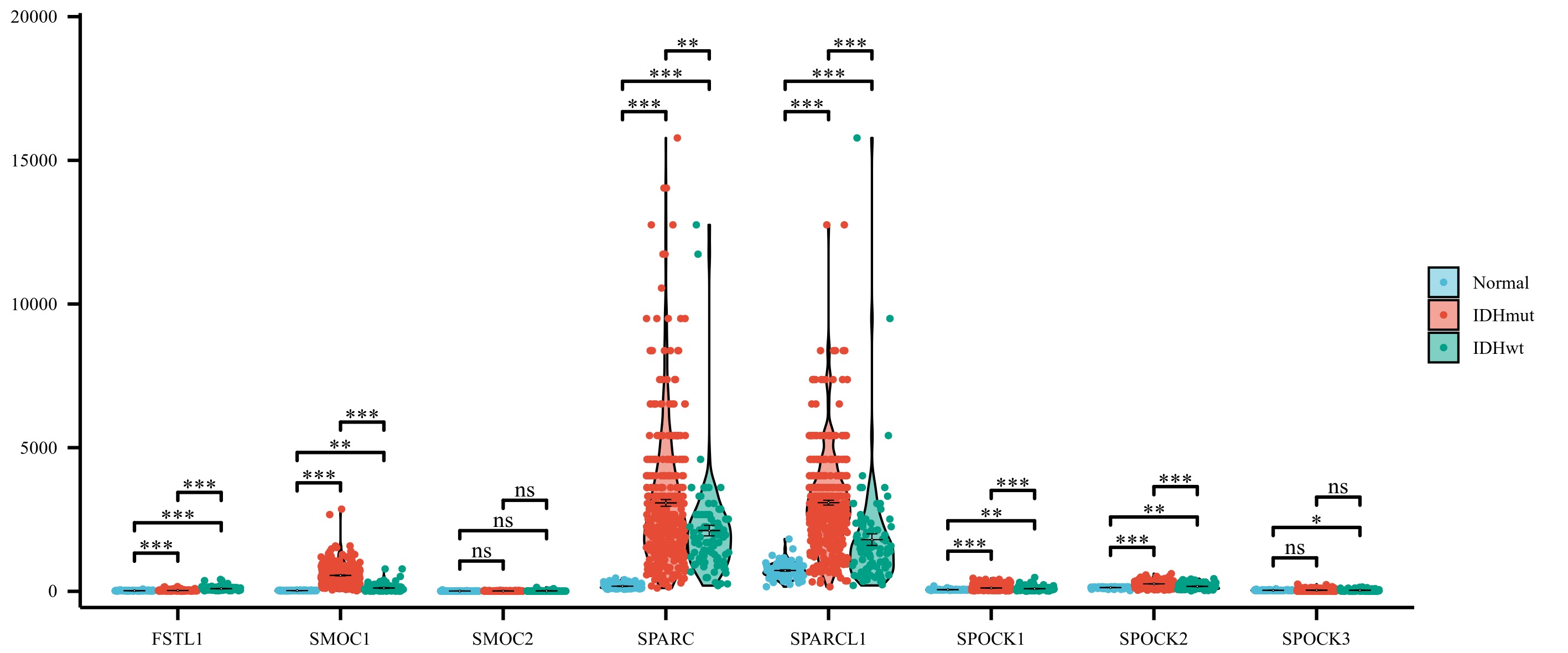

Supplement: Supplementary file 1 — Figure S1. Differential expression of SPARC family genes in normal brain tissue, IDH‐mutant and IDH‐wild‐type gliomas. Statistical analysis was performed using the Kruskal‐Wallis test. *p < 0.05, **p < 0.01, ***p < 0.001. ns, not significance. [file CNR2-8-e70307-s001.jpg]
